# Recent developments in salivary gland pathology after the WHO 2024 classification: new developments in existing entities and evolving new entities

**DOI:** 10.1007/s00428-026-04532-z

**Published:** 2026-04-25

**Authors:** Alena Skálová, Jan Laco, Lester D. R. Thompson, Martina Bradová, Vincent Vander Poorten, Anna Luíza Damaceno Araújo, Göran Stenman, Ilmo Leivo, Abbas Agaimy, Alfio Ferlito

**Affiliations:** 1 Department of Pathology, Faculty of Medicine in Plzen, Charles University, Plzen, Czech Republic; 2 Bioptic Laboratory, Ltd, Plzen, Czech Republic; 3 The Fingerland Department of Pathology, Charles University, Faculty of Medicine and University Hospital Hradec Kralove, Hradec Kralove, Czech Republic; 4 Head and Neck Pathology Consultations, Woodland Hills, CA USA; 5 Otorhinolaryngology, Head and Neck Surgery, Department of Oncology, Section Head and Neck Oncology, University Hospitals Leuven, KU Leuven, Leuven, Belgium; 6 Multidisciplinary Salivary Gland Society, Geneva, Switzerland; 7 Hospital Israelita Albert Einstein, São Paulo, Brazil; 8 Department of Laboratory Medicine/Pathology, Sahlgrenska Center for Cancer Research, University of Gothenburg, Sahlgrenska University Hospital, Gothenburg, Sweden; 9 Institute of Biomedicine, Pathology, University of Turku and Department of Pathology, Turku University Hospital, Turku, Finland; 10 Institute of Pathology, University Hospital Erlangen, Friedrich‐Alexander University Erlangen‐Nürnberg (FAU), Erlangen, Germany; 11 Comprehensive Cancer Center, European Metropolitan Area Erlangen-Nuremberg (CCC ER-EMN), Friedrich Alexander University of Erlangen-Nuremberg, Erlangen, Germany; 12 Coordinator of the International Head and Neck Scientific Group, Padua, Italy

**Keywords:** Salivary gland tumor, World Health Organization classification, Gene fusion, Novel subtypes, Emerging salivary gland tumor entities

## Abstract

Parallel to and after publication of the WHO 2024 classification of head and neck tumors, several developments concerning known existing salivary gland tumor entities, but also proposing new evolving tumor entities have been published. This review article describes the most important new developments in salivary gland pathology published through 2022–2025, that were not included in the 5th edition of the WHO Classification of Head and Neck Tumours 2024. This review summarizes these recent developments in both the benign and the malignant tumor categories. Among the recently proposed entities are palisading adenocarcinoma, microcribriform adenocarcinoma, fenestrating adenocarcinoma and skin-analogue poroid carcinoma. Developments in existing carcinoma entities include recognition of mucoacinar carcinoma as subtype of mucoepidermoid carcinoma (*MAML2*-fused), mucoepidermoid carcinoma without squamous cell differentiation, metatypical adenoid cystic carcinoma, and adenoid cystic carcinoma with prominent tubular hypereosinophilia. In the benign tumor category, recognition of pleomorphic adenoma with canalicular/trabecular phenotype driven by *HMGA2* fusions, triphasic basal cell adenoma with S100 protein-positive "stroma", characterized by *CTNNB1* mutations, metaplastic Warthin tumor with *KRAS* mutations and delineation of thymus-like phenotype in non-sebaceous lymphadenoma with recurrent *CYLD* mutations are the main highlights. Emerging concepts include benign tumor with ductal and papillary morphology (sialadenopapillary ductal tumor). Finally, new grading schemes have been developed/ proposed for acinic cell carcinoma and secretory carcinoma.

## Introduction

The World Health Organization (WHO) Classification of Head and Neck Tumours published the printed 5th edition in 2024 [1]. There were new entities, emerging entities, and significant updates to the taxonomy and characterization of tumors and tumor-like lesions. The major and minor salivary glands are associated with a remarkable diversity of neoplasms. Given the number of already existing salivary gland tumor entities which display overlap of histologic and immunohistochemical features between different entities, only very well documented new entities were accepted in the 5th edition of the WHO classification [1]. Recognition of intercalated duct adenoma (in addition to hyperplasia), striated duct adenoma and sclerosing polycystic adenoma (not adenosis) were the main changes in the benign category. In the malignant tumor category, mucinous adenocarcinoma, microsecretory adenocarcinoma and sclerosing microcystic adenocarcinoma were the new-comers. Salivary gland tumor taxonomy, however, has expanded further since the publication of the WHO classification, with introduction of several novel subtypes of established entities, such as mucoacinar subtype of mucoepidermoid carcinoma (MEC) [2], metatypical adenoid cystic carcinoma (AdCC) [3], and AdCC with striking tubular eosinophilia [4], with delineation of novel entities and introduction of analogous new concepts. In previous editions of the WHO Classification, new entities have been included, such as secretory carcinoma in the 4th edition [5, 6] and microsecretory adenocarcinoma in the 5th [7]. After the Editorial Board Meeting of the 5th edition, novel entities such as microcribriform adenocarcinoma [8] and palisading adenocarcinoma [9] have been described, but they are not included in the WHO Classification yet due to incomplete characterization. Many of these new developments depend on identifying various molecular genetic alterations, failure to find any genetic abnormality should not, however, invalidate conventional histological and immunohistochemical evaluation.

## Newly described (non-WHO-listed) salivary gland tumors

### Palisading adenocarcinoma

Palisading adenocarcinoma (PaA) was first described by Bishop et al. in 2023 in a series of nine cases, consisting of eight females and one male aged 45–74 years (median 57 years) [9]. In seven cases these tumors were localized in the sublingual gland, which is a rare site for a salivary gland tumor [10]. The remaining two tumors affected the submandibular gland. The prognosis of PaA seems favorable during the reported follow-up period (range 4–160 months; median 53 months), without any patient experiencing local recurrence or metastasis.

Since the first description of PaA, several case reports have been published confirming the originally described characteristics of this tumor, i.e. its higher incidence in females and its frequent involvement of the sublingual and submandibular glands [11, 12]. A recent study describes PaA in six females within one family, with each patient having two or more tumors [13]. Furthermore, the localization of most tumors in the parotid gland was novel, but unexplained. This new study also confirmed the significantly higher incidence of PaA in females of Asian ethnicity.

Although the course of the disease so far is described as favorable, the biological nature of the tumor, i.e. whether it is always a low-grade adenocarcinoma, remains uncertain. It is necessary to wait for data from longer follow-ups in larger future studies.

**Fig. 1:**
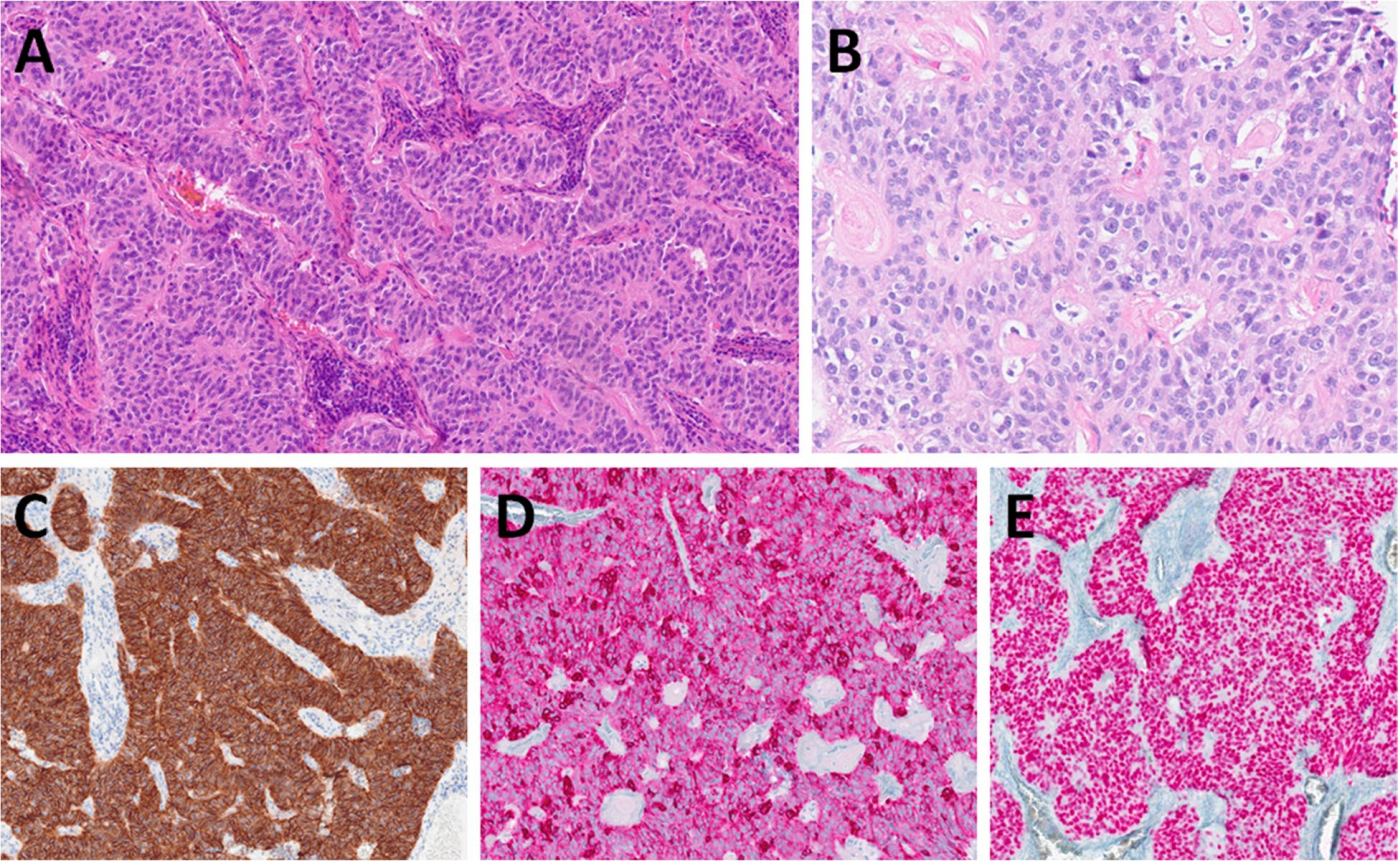
Palisading adenocarcinoma is composed of biphasic trabeculae consisting of polygonal cells with prominent nucleoli and pale eosinophilic cytoplasm (**A**). The tumor cells may focally form palisades or pseudorosettes around hyalinized stroma or blood vessels (**B**). Tumor cells are positive for CD56 (**C**) and pancytokeratin, including CAM5.2 (**D**). Androgen receptor expression is seen in most cases (**E**)

Microscopically, PaA shows a biphasic appearance (Fig. 1A). The predominant tumor cell population consists of trabecularly arranged polygonal cells with round nuclei, prominent nucleoli and a weakly eosinophilic cytoplasm. The tumor cells can focally form palisades or pseudorosettes around hyalinized stroma or vessels (Fig. 1B). Immunohistochemically, the tumor cells diffusely express CD56 (Fig. 1C) and are variably reactive with a cocktail of cytokeratin antibodies (CK) (Fig. 1D), and antibodies to S100 protein and androgen receptor (Fig. 1E). Despite a neuroendocrine-like appearance and a strong CD56 immunopositivity, specific neuroendocrine markers, such as chromogranin, synaptophysin and INSM1 are typically negative. Sprinkled within this predominant tumor cell population, although difficult to detect, are small ducts. These ducts are strongly reactive for CK cocktail, CK5/6, and CK7 antibodies (and sometimes also for SOX10, DOG1 and CD117 antibodies), but are unexpectedly CD56 negative. Lymphatic invasion and perineural spread are occasionally noted. Despite through molecular profiling, no characteristic oncogenic driver mutation or fusion has been found, although the reported familial cases argue for possible inherited yet unknown mutation in that family [9, 13].

### Microcribriform adenocarcinoma

Microcribriform adenocarcinoma (MiA) is closely related to microsecretory adenocarcinoma (MSA). The index case of MiA was initially included in the microsecretory adenocarcinoma series by Bishop et al. [7], but then delineated as morphologically and genetically distinct entity in a series of four cases published in 2023 [8], showing a unique morphology, immunoprofile, and a tumor type-specific gene fusion *SS18*::*ZBTB7A* in contrast to the *MEF2C*::*SS18* as a marker of microsecretory adenocarcinoma [7, 8].

MiA affected three females and one male, with an age range of 24–65 years (median 60 years). Two tumors involved the parotid gland, one the submandibular gland and one was located in minor glands of the bronchial mucosa. Clinical data were available for two patients with follow-up periods of 66 months and 12 months, respectively. No patient experienced locoregional or distant recurrence after tumor resection followed by adjuvant radiotherapy. Since no further cases of MiA have been published, a reliable assessment of the prognosis of this very rare malignant salivary gland tumor is not yet possible.

**Fig. 2:**
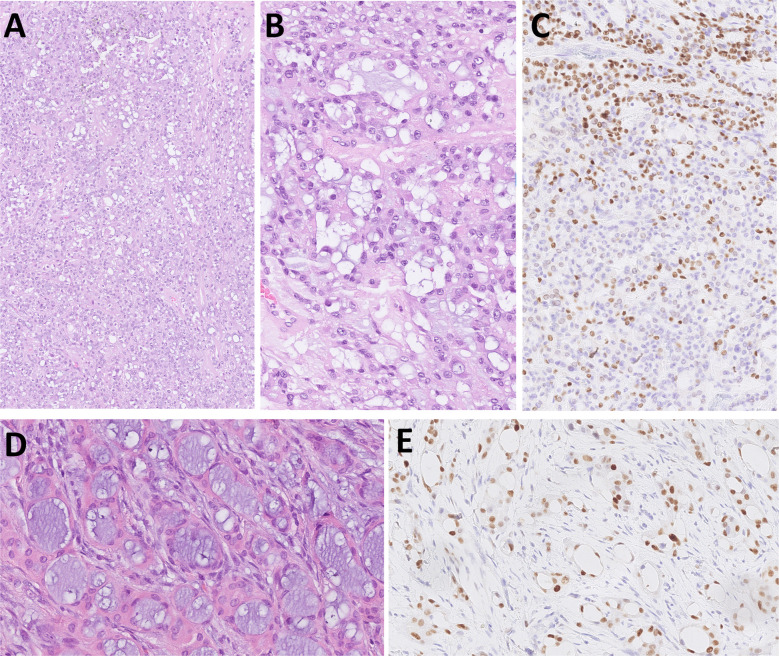
Microcribriform adenocarcinoma is composed of cribriform, tubular and solid architecture separated by fibrous stroma (**A**). Plenty small spaces called “microcribriform structures” are present, and some tumor cells may show oncocytoid appearance (**B**) and focal immunoexpression for p40 (**C**). Microsecretory adenocarcinoma is architecturaly more variable with a predominant microcystic growth pattern, bluish luminal secretions and fibromyxoid stroma (**D**) and is typically p63 positive (**E**)

Microscopically, the tumor is composed of cribriform, tubular, or solid growth patterns, which additionally create multiple small spaces “microcribriform” structures (Fig. 2A, B). Some tumor cells may show an oncocytic change of the cytoplasm. Compared to MSA (Fig. 2D), MiA is architecturally and cytologically more variable, and secretory features are less pronounced. The tumors occasionally show infiltrative growth within a hyalinized and myxoid stroma. Immunohistochemically, the tumor cells express CKs, S100 protein, and SOX10 and only focally p63, p40, calponin and SMA. MSA and MiA share immunoreactivity for S100 protein and SOX10 but differ in their expression of p63 and p40 (Fig. 2C). While MSA is characterized by diffuse p63 positivity and complete absence of p40 (Fig. 2E), MiA shows an absence of both markers, although a focal reactivity in the myoepithelial component of the tumor may be seen. Next generation sequencing (NGS) can be used to unambiguously differentiate MiA from MSA, as these tumors harbor the distinct canonical fusions *SS18*::*ZBTB7A* and *MEF2C*::*SS18*, respectively.

### New subtypes or patterns in already established salivary gland neoplasms

In this section, the following newly described subtypes/variant morphologies of well-defined and established malignant salivary gland tumors are discussed: mucoacinar carcinoma and mucoepidermoid carcinoma (MEC) without morphologically distinct squamous cell differentiation as new subtypes of MEC, and metatypical AdCC and AdCC with prominent tubular hypereosinophilia.

### Mucoacinar carcinoma

MEC is one of the most common malignant salivary gland tumors, arising in both minor and major salivary glands. MEC is characterized by mucous (mucin-producing), intermediate, and squamoid cells [1]. Architectural configurations include cystic and solid areas where proportions of tumor cell types vary widely. Cystic spaces are lined partly by mucous cells with abundant mucinous cytoplasm and peripherally situated nuclei, and they display intracytoplasmic mucicarmine or PAS staining with diastase resistance. Extracellular mucin may be present. Intermediate cells are often the most frequent tumor cell type. Significant keratinization is exceptional. Cell populations with clear, columnar, or oncocytic cells may be present, and occasionally they predominate. Although the diagnosis of MEC is easy in most cases, recognition of its less common subtypes can be difficult. The current WHO classification lists seven growth patterns/subtypes of MEC, including sclerosing [14], clear cell [15], oncocytic [16], Warthin-like [17], ciliated [17], spindle cell [18], and mucoacinar [2].

**Fig. 3:**
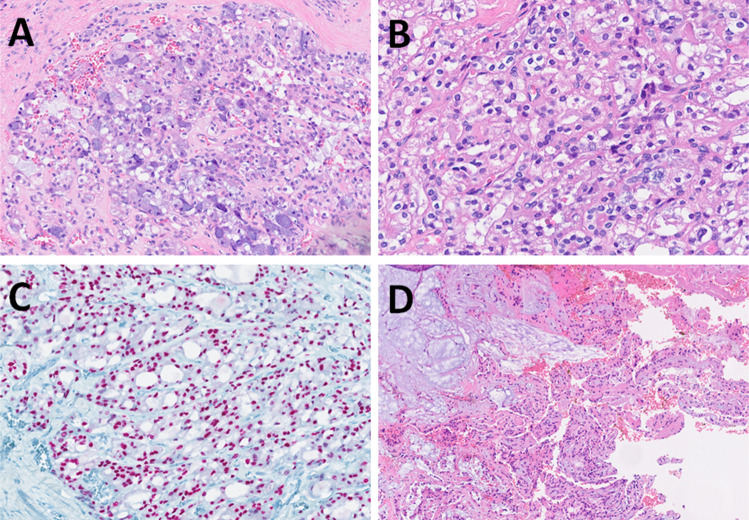
Mucoacinar carcinoma is usually solid (**A**), composed of a variable spectrum of tumor cells including mucinous, acinar, and clear cells (**B**). Tumor cells are typically positive for SOX10 (**C**). Occasionally, extracellular mucin leakage may be present (**D**)

Mucoacinar carcinoma was first described in 2021 in a series of 11 cases of MEC with simultaneous differentiation into serous acinar cells (Fig. 3A) [2]. The series consisted of seven females and four males aged 21–72 years (median 55 years). Ten tumors involved the parotid gland and one tumor occurred in the submandibular gland. Three tumors were grade 1, seven were grade 2, and one was grade 3. Clear cell change was often observed in grade 1 and 2 tumors (Fig. 3B). Serous acinar tumor cells constituted 5–10% of the tumor. Morphologically evident serous acinar differentiation with PAS-D positivity was further confirmed by immunohistochemistry for SOX10 (9/9 cases) (Fig. 3C) and DOG1 (9/10 cases), with NR4A3 expression in 6/7 cases (although focal and corresponding mostly to serous acinar tumor cells). These findings are typical of acinic cell carcinoma (AciCC). The other component of the tumors contains, however, mucocytes and extracellular mucin (Fig. 3D). Using fluorescent in situ hybridization (FISH), the *MAML2* gene rearrangement, typical of MEC, was demonstrated in all cases, while *NR4A3* or *MSANTD3* gene rearrangements, typical of AciCC, were not detected.

The differential diagnosis of mucoacinar carcinoma is significantly complicated by the fact that a squamoglandular subtype of AciCC has recently been described [19]. This case report of a parotid gland tumor in a 59-year-old male shows a tumor morphology essentially consistent with mucoacinar carcinoma, including immunohistochemical expression of DOG1 and NR4A3 markers. However, FISH demonstrated a rearrangement of the *NR4A3* gene typical of AciCC, while evidence for rearrangement of the *MSANTD3* and *MAML2* genes was negative. Furthermore, there is a case report of a left parapharyngeal space tumor in an 81-year-old male describing a case of AciCC with high-grade squamoglandular and chondrosarcomatous transformation [20]. In immunohistochemistry, the tumor cells expressed NR4A3 and were negative for PLAG1 and HMGA2. This immunoprofile was consistent with the FISH result, which demonstrated a *NR4A3* gene rearrangement, while the *PLAG1* and *HMGA2* genes were intact [20].

These cases demonstrate the importance of performing molecular genetic testing in salivary tumors with unusual morphologies. At the same time, the paradigm that any malignant salivary gland tumor with serous acinar differentiation must necessarily be AciCC is no longer valid. Still, in large series, such unique cases are rare: studies indicate that the diagnostically difficult cases described above are fortunately encountered only rarely. Nakano, et. al., did not record a single case of mucoacinar carcinoma in their study of 177 cases of MEC [21]. Nevertheless, it is important to be aware of this diagnostic challenge.

### Mucoepidermoid carcinoma without morphologically distinct squamous cell differentiation

The name of this subtype of MEC seems at first glance to be a contradiction, since MEC is defined as “a malignant tumor characterized by solid and cystic patterns with mucinous (mucin-forming), intermediate, and epidermoid cells, most often associated with *MAML2* gene rearrangement” [1]. However, a recent publication shows that squamous differentiation can be absent in unequivocal MEC, both histologically and immunophenotypically, suggesting that an update in the definition of MEC is needed [22].

The first observation of this interesting subtype was published in 2023 by Bishop et al., who described 10 cases of MEC, none of which showed squamous differentiation on classical HE staining, nor any classical markers of squamous cell differentiation, i.e., CK5/6, p63, or p40 [22]. The patients consisted of eight females and two males with an age range of 9–84 years (median 40 years). The tumors most often affected the parotid (four cases) and submandibular glands (two cases). The remaining tumors originated from the minor glands of the base of the tongue, the nasopharynx, bronchi and the trachea. Six tumors were assessed as low-grade, two as intermediate-grade, one as high-grade, and one low-grade tumor showed high-grade transformation (formerly dedifferentiation). In hematoxylin and eosin staining, distinct squamous cell differentiation was not observed in any of the tumors, but nine of the ten tumors were classified into one of the known subtypes of MEC. Four tumors were classified as oncocytic subtype, three as clear cell subtype, one as spindle cell subtype, and one as a combined clear cell and spindle cell subtype. The last tumor (a high-grade MEC) showed unusual solid and micropapillary growth.

Despite negative immunohistochemistry for CK5/6, p63 and p40, the diagnosis of MEC was confirmed in all cases by molecular genetic testing. Seven cases had a known canonical fusion (*CRTC1*::*MAML2* in five cases and *CRTC3*::*MAML2* in two cases). In the high-grade MEC with unusual morphology, a novel *MAML2*::*CEP126* fusion was demonstrated. In the remaining two cases, RNA sequencing was not possible due to lack of material, but a FISH break-apart probe demonstrated a *MAML2* gene rearrangement. In 2023, Ahn, et. al. published seven MECs with unusual solid or trabecular morphologies that showed only weak and focal, or completely absent expression of p63, while CK5/6 and p40 were not examined in this study [23]. This study included five females and two males with an age range of 26–55 years (median 44 years). Three tumors affected the parotid gland, two affected the tongue root, and one each the hard palate and the parapharyngeal space. The diagnosis of MEC was confirmed in all cases by detection of a *MAML2* gene break using FISH, and in three cases the *CRTC1*::*MAML2* fusion was detected by RNA sequencing.

Based on these observations, it has been proposed to modify the definition of MEC: “MEC is a carcinoma with conventional features (mucinous, intermediate, squamous cells) and/or with evidence of *MAML2* gene rearrangement”. It remains to be seen whether this proposal will be adopted in the next edition of the WHO classification of head and neck tumors.

### Metatypical adenoid cystic carcinoma

AdCC is a biphasic destructively invasive carcinoma composed of epithelial and myoepithelial neoplastic cells arranged in tubular, cribriform, and solid patterns associated with basophilic matrix and reduplicated basement membrane material, and often with *MYB* or *MYBL1* gene rearrangement [1].

In 2022, Matthew, et al. described three cases of AdCC of the skull base, which showed an unusual morphology [3]. The patients were two males and one female aged 40–60 years. Microscopically, in addition to cribriform areas formed by basaloid cells, the tumors also displayed areas of trabecular and macrocystic growth, and areas with squamous cell differentiation. Immunohistochemically the tumor cells expressed, among others, CK5/6, p63, p40 and focally c-kit and p16. Molecular genetic examination demonstrated a *MYB*::*NFIB* fusion in two cases and a *MYBL1*::*NFIB* fusion in one case, i.e., the molecular genetic features typical of AdCC.

**Fig. 4:**
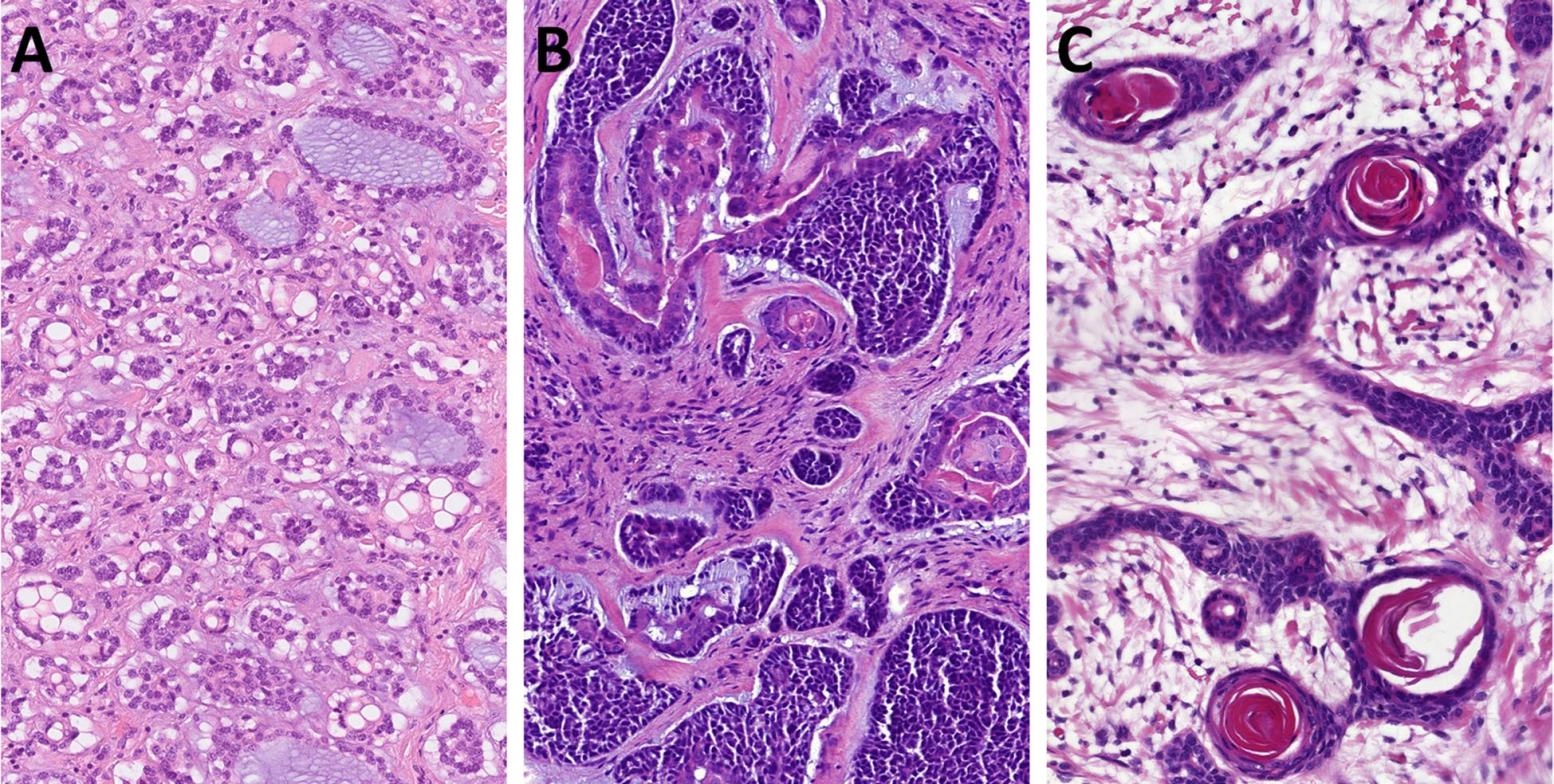
Metatypical adenoid cystic carcinoma may be composed predominantly of vacuolated cells (**A**), can show tubular hypereosinophilia of luminal cells (**B**), and may even exhibit squamous formations (keratin pearls) (**C**)

In 2023, Ooms, et. al. published a case report of a 72-year-old male with a recurrent metatypical AdCC of the nasal cavity and the maxillary sinus, which also showed sebaceous differentiation [24]. The diagnosis of AdCC was supported by demonstration of a *MYB*::*NFIB* gene fusion. In 2025, another case report of a 68-year-old male with metatypical AdCC, involving the minor salivary glands of the hard palate was reported [25]. Metatypical AdCC is a rare subtype of this tumor. In a recent study, Skálová et al. identified five cases of metatypical AdCC of the sinonasal tract in an analysis of a total of 88 cases of sinonasal AdCC (6%) [26]. It is noteworthy that three of these cases were found to have non-canonical fusions, such as *EWSR1*::*DNM3, EWSR1*::*MYB* and *ACTN4*::*MYB.* In addition, it has been reported that the prognosis of patients with metatypical AdCC with a *NOTCH* or *BCOR* gene mutation is extremely unfavorable [26]. Metatypical AdCC is an extension in the differential diagnosis of tumors with squamous cell differentiation in the head and neck, which must now also include this rare tumor (Fig. 4A, B, C).

### Adenoid cystic carcinoma with striking tubular hypereosinophilia

AdCC with striking tubular hypereosinophilia is another subtype of AdCC that is not formed by classic basaloid cells with hyperchromatic angulated nuclei. This subtype of AdCC was described in 2023 in a series of 16 cases involving eight males and eight females aged 33–89 years (median 66 years) [4]. None of these tumors involved the major salivary glands, instead all of them arose from minor mucosal salivary glands in the following locations: the sinonasal tract, the larynx, the trachea, the external auditory canal, the nasopharynx, the base of the tongue, the hard palate, and the floor of the mouth. Their microscopic growth patterns included, in addition to the classical architecture (tubular, cribriform, solid), also glomeruloid, micropapillary, and morular growth patterns. A striking feature of all such tumors was the presence of large ductal luminal tumor cells with abundant eosinophilic cytoplasm and resemblance to Paneth cells. In addition to the canonical *MYB*::*NFIB* and *MYBL1*::*NFIB* fusions, molecular genetic examination revealed novel *EWSR1*::*MYB* and *FUS*::*MYB* fusions, which were not present in any of the 102 classical AdCCs examined as a control group.

AdCC with prominent tubular hypereosinophilia is undoubtedly a rare tumor, since only one other case report in a 52-year-old female with a tumor affecting the floor of the mouth [27] has been published, while 14 additional cases were identified in a study of 88 sinonasal AdCCs [28].

For correct microscopic diagnosis, it is important to know that AdCC does not always have classic morphology, but that there are (albeit rare) subtypes with very different morphologies. Furthermore, the spectrum of molecular genetic alterations in AdCC is also expanding, as novel variant fusions have been described, e.g. *TULP4::MYB, ACTB*::*MYB* and *ESRRG::DNM3* [29].

Finally, it is curious that some AdCCs of the sinonasal tract may arise from or in association with respiratory epithelial adenomatoid hamartoma (REAH) or seromucinous hamartoma [28, 30], suggesting potential precursor lesions of sinonasal AdCC. Furthermore, the description of five cases of sinonasal adenosquamous carcinoma arising in association with these hamartomas supports roles for them in malignant transformation [31].

### Salivary carcinosarcoma (sarcomatoid carcinoma ex pleomorphic adenoma)

**Fig. 5:**
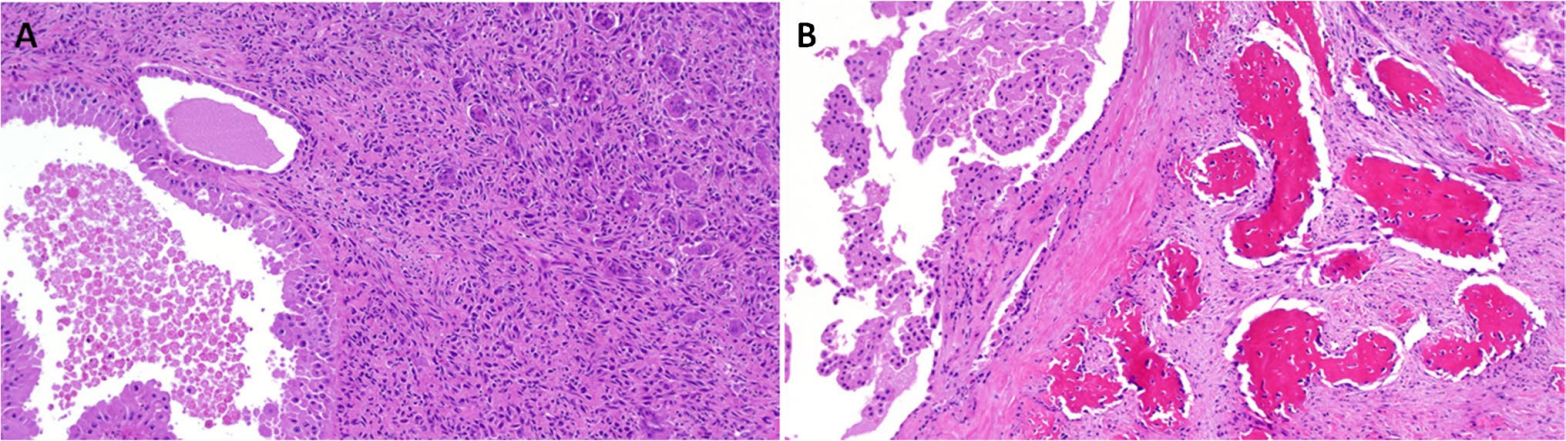
Carcinosarcoma (sarcomatoid salivary duct carcinoma) without (**A**) or with heterologous mesenchymal osteosarcomatous elements (**B**)

Although the “carcinosarcoma” terminology was kept in the WHO 2024 classification, parallel evidence has shown that these tumors are indeed analogous to their counterparts in other organs. Notably, most salivary carcinosarcomas originate from preexistent PA and display an epithelial component (salivary duct carcinoma in most cases followed by myoepithelial carcinoma) associated with an additional sarcoma-like mesenchymal component. The latter may display either undifferentiated pleomorphic phenotype (undifferentiated pleomorphic sarcoma-like) or show specific mesenchymal line of differentiation, mostly osteogenic or chondroblastic. Notably, clonal origin could be verified in most cases, indicating origin as sarcomatoid pattern of carcinoma ex PA [32]. Based on these data, the term “sarcomatoid carcinoma (including exact subtype) with or without heterologous mesenchymal elements” might be more appropriate than the old terminology “carcinosarcoma” (Fig. 5A-B) [32].

### Recent developments in benign and low-grade tumor category

#### Pleomorphic adenomas with HMGA2 alteration and canalicular/trabecular histotype

**Fig. 6:**
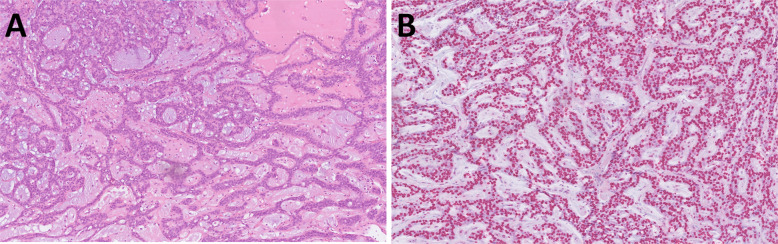
Canalicular pleomorphic adenoma is often composed of multilayered, interconnecting and branching strands and trabeculae, with minimal or only focal chondromyxoid stromal component. **A** Tumor cells are positive for HMGA2 surrogate marker (**B)**

Pleomorphic adenoma (PA), the most common salivary gland tumor, is driven by chromosomal rearrangements involving *PLAG1* (mapped to 8q12) or *HMGA2* (mapped to 12q14-15) in most cases [33]. Multiple fusion partners have been identified including *CTNNB1, FGFR1, LIFR, CHCHD7* and *TCEA* for *PLAG1* fusions and *NFIB, WIF1* and FHIT for *HMGA2* fusions [1, 34]. Agaimy et al. reported a study of 28 major salivary gland tumors displaying distinctive trabecular and canalicular morphology associated with a recurrent *HMGA2*::*WIF1* fusion [35]. The patients were 15 females and 13 males aged 43 to 87 (median: 65). All tumors originated from the parotid gland. Their size ranged from 10 to 40 mm (mean: 23 mm). Histologically, all tumors showed elongated or columnar cells arranged into bilayered or multilayered communicating and branching strands and trabeculae in a manner similar to canalicular adenoma of minor salivary glands or trabecular myoepithelioma with variable solid confluent intercalated duct-like areas (Fig. 6A) and expressed nuclear HMGA2 immunopositivity (Fig. 6B). Fifteen tumors were exclusively canalicular/trabecular while 13 had an intermingled or well-demarcated conventional (chondromyxoid) PA component comprising 5 to > 50% of the tumor [35]. Histological, immunohistochemical and molecular features of the *HMGA2*-positive subtype of pleomorphic adenoma with prominent trabecular/canalicular morphology have been further confirmed in subsequent studies [36–39]. Carcinoma ex pleomorphic adenoma has been described to occur in this phenotype of PA [36, 39].

#### Triphasic basal cell adenoma with S100 protein-positive "stroma” and *CTNNB1* mutation

**Fig. 7:**
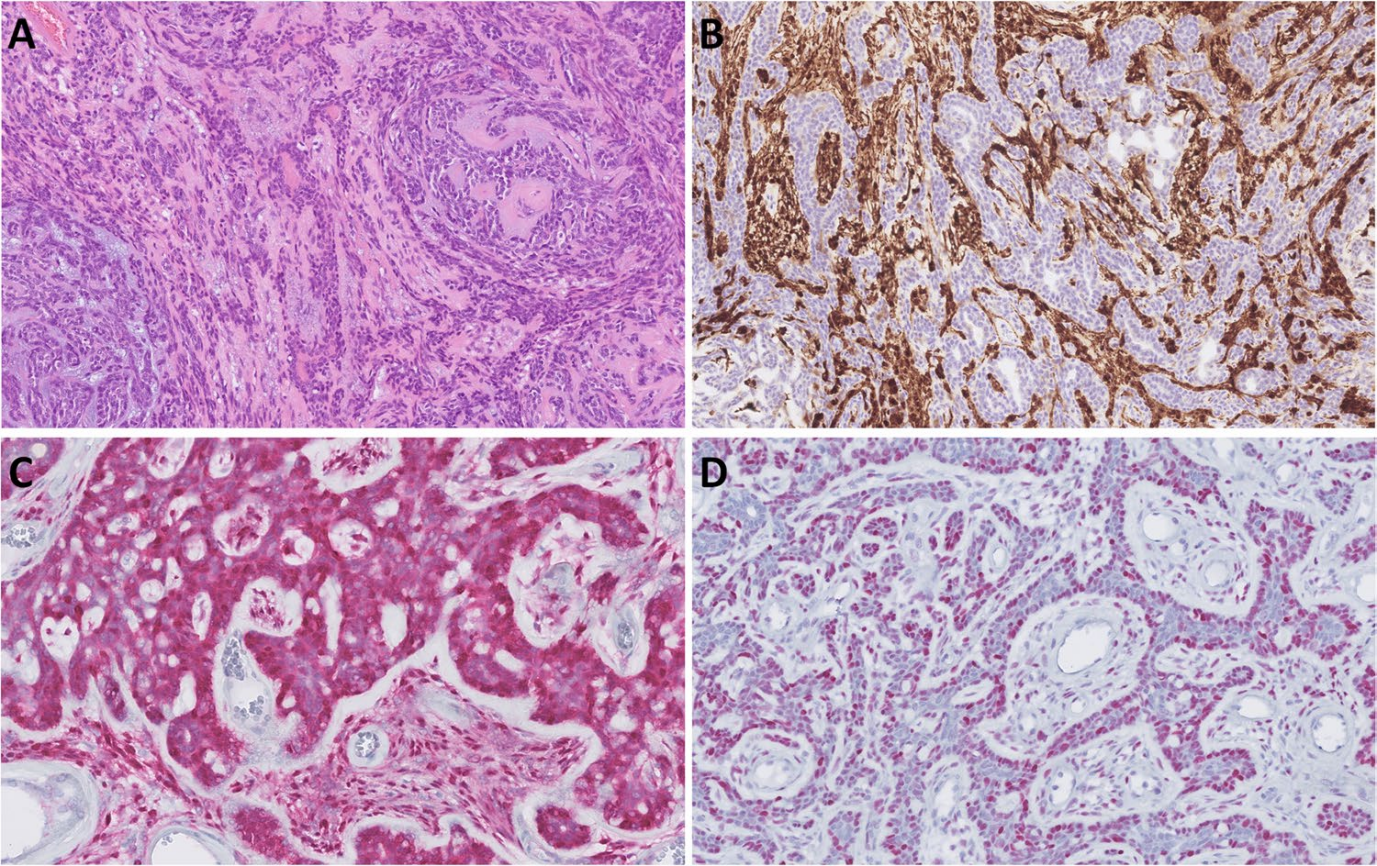
Basal cell adenoma with S100 protein-positive stromal cells is therefore a triphasic tumor composed of luminal and abluminal epithelial tumor cells together with neoplastic spindle-shaped stromal cells (**A**). The latter, and occasionally the abluminal cells, are positive for S100 protein (**B**). Notably, nuclear β-catenin (**C**) and LEF1 (**D**) are positive even in all types of cells

Basal cell adenoma (BCA) is a benign biphasic salivary gland neoplasm composed of basaloid and luminal cells and often containing basement membrane material [1]. BCAs are encapsulated or well circumscribed and show tubular, trabecular, cribriform, membranous, or solid growth. The tumors show peripheral palisading of dark cells with luminal paler cells and ducts. A subset of BCA shows a distinct S100 protein-positive stromal component of spindled cells and thus morphologically displays triphasic differentiation, where the neoplastic spindle-shaped stromal cells are found along a bilayered neoplastic epithelium (Fig. 7A-D) [40]. Recently, two studies focusing on a S100 protein-positive stroma in salivary gland BCAs were published in *Virchows Archiv* [41, 42]. Skálová et al. investigated the classification of BCA with S100-protein positive stroma by comparing it with BCA without S100 protein-positive stroma and with PAs using DNA methylation profiling. Methylation analysis revealed that BCA with S100 protein-positive stroma forms a distinct cluster, separate from both PA and classic BCA which lacks both the S100 protein-positive stroma and *CTNNB1* mutations [41]. These results suggest that identifying a S100 protein-positive stroma in salivary gland tumors not only aids in distinguishing BCA from other histological types but may also have implications for subclassification within BCA itself [41].

Sasaki et al. confirmed the presence of identical *CTNNB1* mutations in both the stromal component and the epithelial/myoepithelial component using manual microdissection combined with digital polymerase chain reaction [42]. This molecular evidence strongly supports the neoplastic nature of the S-100 protein positive stromal cells. Collectively, these findings suggest that BCAs can exhibit tricellular differentiation with presence of neoplastic spindle-shaped stromal cells alongside a bilayered neoplastic epithelium, and that such BCAs are characterized by frequent *CTNNB1* mutations [43].

#### Proliferating (metaplastic) Warthin tumor with *KRAS* mutation

**Fig. 8:**
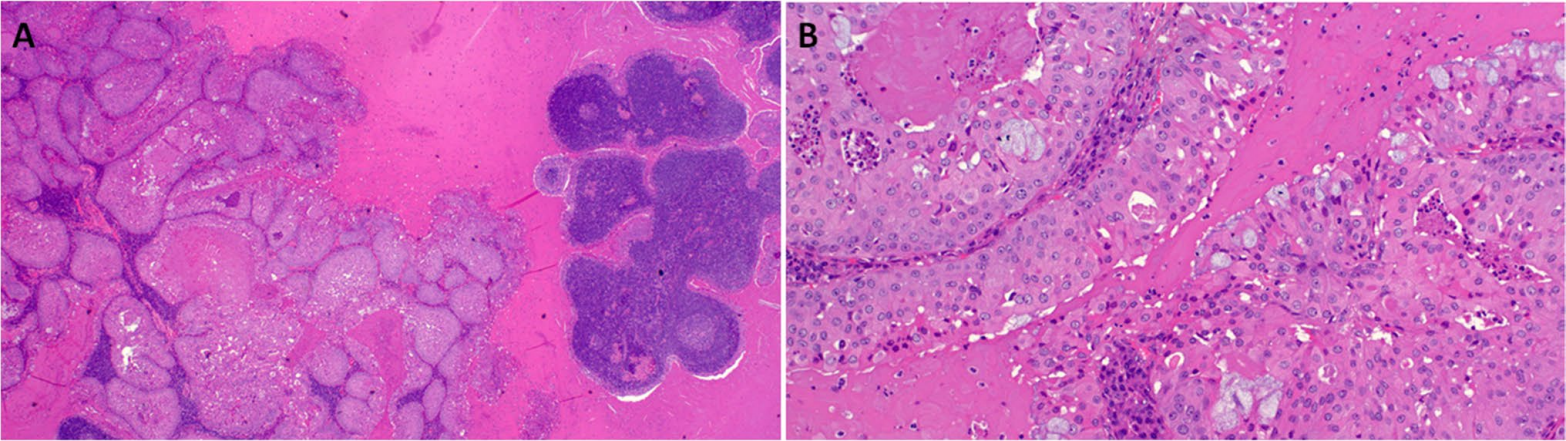
*KRAS*-mutated de novo proliferating squamoid Warthin tumor reveals a solid and straified epidermoid proliferations (**A**) with variable mucinous cells, mimicking mucoepidermoid carcinoma (**B**)

Warthin tumor (WT) is a benign epithelial tumor of salivary glands that occurs almost exclusively in the parotid gland and its associated lymph nodes [1]. WT represents the second most common type of salivary gland tumors [44]. Histologically, the tumor displays tall columnar oncocytic cells arranged in two cell-thick layers lining variably cystic spaces within a lymphoid stroma [1]. Tumors with exuberant squamous metaplasia in response to FNA-induced or other types of tissue injury/infarction have been referred to as “metaplastic WTs” [45]. However, the same terminology is used for tumors with variable mucinous cell and solid or stratified epidermoid proliferations occasionally mimicking MEC (Fig. 8A-B), although the “concept of metaplasia” has never been proven for the latter [46]. In a recent study, WTs showing prominent mucoepidermoid-like or solid oncocytoma-like proliferations without prior FNA or histological evidence of infarction/trauma were investigated, and an oncogenic *KRAS* mutation at codon 12 was found in 27% of these tumors, while all conventional WTs lacked such mutation [46]. This finding supports a neoplastic nature for the epidermoid/mucoepidermoid proliferations in non-injured “metaplastic” WTs. Therefore, the descriptive term “de novo proliferating Warthin tumor” was proposed for this subtype to distinguish it from infarcted/inflamed genuine metaplastic WTs [46].

## Emerging salivary gland tumors

### Sialadenopapillary ductal tumor unifying the spectrum of sialadenoma papilliferum-like tumors

Sialadenoma papilliferum (SP) is a polypoid neoplasm of mucosal surface epithelium and salivary duct epithelium, characterized by the presence of both an exophytic surface squamous cell and endophytic ductal papillary proliferations [1]. The exophytic squamous cell proliferation is directly contiguous with submucosal ductal epithelium that shows endophytic papillary infoldings and cyst-like spaces. A *BRAF* p.V600E mutation is consistently reported in SP [47]. However, there are cases in which the surface epithelial involvement/exophytic growth is absent. In their study, Nakaguro et al. [48] used the term “sialadenoma papilliferum-like intraductal papillary tumor” (SP-IPT) for tumors that are morphologically similar to SP, but lack the surface epithelial component. Their cases of SP-IPT demonstrated the *BRAF* p.V600E mutation, with one case showing an *HRAS* p.Q61R co-mutation [48]. Both of these tumors bore similarities to cutaneous adnexal tumors, such as syringocystadenoma papilliferum, tubular adenoma (also called tubulopapillary hidradenoma), and papillary eccrine adenoma. So-called SP, SP-IPT, and tubulopapillary hidradenoma-like tumor of the mandible [49] all share morphologic and molecular similarities, with no reproducible distinguishing features and a potential for locally aggressive growth and regional metastasis. Given the lack of reliable ways to differentiate between these tumors, it seems that using a unifying term to reflect their common morphologic and molecular features and low malignant potential is desirable: “sialadenopapillary ductal tumor” [50].

### Skin analogue primary poroid neoplasms of salivary glands with *YAP1/WWTR1::MAML2/NUTM1* fusions

**Fig. 9:**
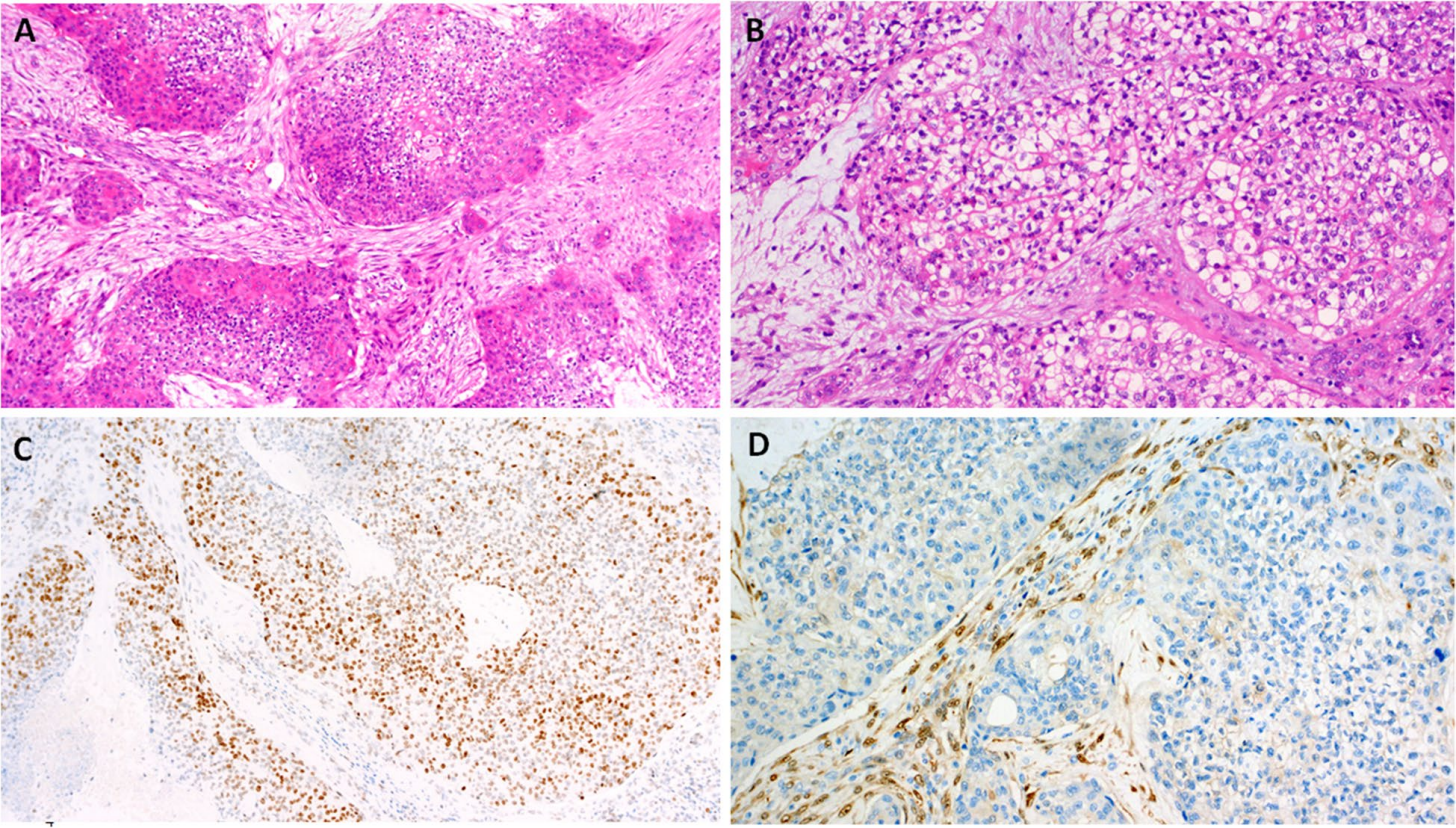
Skin analogue primary poroid carcinoma of parotid showing multiple large lobules with variable squamoid eosinophilic and clear cells with eosinophilic cutical cells at periphery surrounding basophilic poroid cells (**A**) and higher magnification of the clear cell areas (**B**). Representative immunohistochemical findings show diffuse strong expression of p40 (**C**) and loss of YAP1 C-terminus with preserved expression in the background stromal cells (**D**)

Skin analogue poroid neoplasms originating from major and minor salivary glands, that recapitulate the spectrum of cutaneous poroid tumors, were recently introduced as a possible novel salivary gland tumor entity [51]. Some cases have a bland poroma-like morphology, while others are high-grade porocarcinoma-like malignancies [52, 53]. Histologically, cutaneous porocarcinoma displays a variable admixture of monomorphic small basaloid-looking poroid epithelial cells admixed with a variable component of large eosinophilic cuticular cells (Fig. 9A-B) [54]. A variable ductal component may be present, but this is usually a focal finding only, and many tumors lack overt ductal differentiation [55]. In the appropriate clinical and morphological context, the *YAP1/WWTR1*::*MAML2/NUTM1* fusions detected in these tumors are highly specific for poroid neoplasms, both cutaneous and non-cutaneous. This tumor type should be distinguished from the many primary and metastatic salivary neoplasms with immunophenotypically squamous cell differentiation (Fig. 9C-D). In particular, the *NUTM1*-rearranged (NUT-immunopositive) poroid neoplasms should be distinguished from the more aggressive NUT carcinoma, as both tumor types occur in overlapping anatomic sites and show a squamous cell phenotype and are NUT immunoreactive. Moreover, *MAML2*-fused poroid neoplasms might be confused with a subtype of high-grade MECs, as they share a squamous phenotype and *MAML2* rearrangement [56].

### Fenestrating adenocarcinoma with *EWSR1::BEND2* fusion

A possibly novel salivary gland carcinoma with *EWSR1*::*BEND2* fusion with predilection for the pharynx was published recently [57]. This study described a cohort of seven minor salivary gland tumors mostly occurring in the pharynx (four in the base of tongue, one in the pharyngeal wall/tonsil, one nasopharynx, and one in the trachea), showing a unique fenestrated appearance, architectural diversity, and a specific *EWSR1*::*BEND2* gene fusion [57]. All cases tested by NGS showed an *EWSR1*::*BEND2* fusion, as well as immunohistochemical CK7 and BEND2 positivity, and S100 protein negativity. The tumors showed variable p63 staining depending on whether squamous or basaloid features were present. A salivary gland neoplasm with *EWSR1*::*BEND2* fusion was first reported at the base of the tongue, and it showed a varied architecture of bland cells arranged in cribriform-like structures, dyscohesive sheets, and individually infiltrative cells [58]. The tumor cells had eosinophilic and focally vacuolated cytoplasm and some tumor cells showed a signet ring-like appearance [58]. The *EWSR1*::*BEND2* fusion and immunohistochemical BEND2 staining is lacking from other salivary gland neoplasms and may be useful when establishing this diagnosis. The name “fenestrating adenocarcinoma (FAC)” of salivary glands was proposed for this tumor entity [57].

### Thymus-like phenotype in benign lymphoepithelial neoplasms and its relationship to non-sebaceous lymphadenoma

Non-sebaceous lymphadenoma (NSLA) is a rare, benign, well circumscribed lymphoepithelial tumor, first described by Auclair et al. in 1991 [59], and included in the 2005 WHO classification. In the current WHO classification, NSLA and sebaceous lymphadenoma are included under the heading of “lymphadenomas” [1]. Together, they represent 0.1% of all salivary gland neoplasms and < 0.5% of salivary adenomas.

**Fig. 10:**
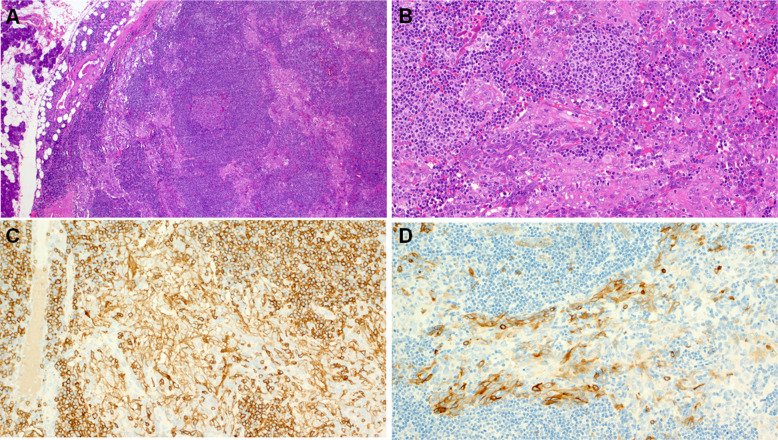
Lymphoepithelial neoplasm with thymus-like phenotype is composed of large squamoid cells with indistinct cell borders, forming large irregular branching and anastomosing aggregates within a lymphoid stroma with Hassall corpuscle-like structures and intraepithelial sprinkling of lymphocytes (**A-B**). All tumors were positive for CD5 (**C**) and CD117 (**D**)

Benign lymphoepithelial tumors of salivary glands have been restricted to sebaceous and non-sebaceous lymphadenomas. However, salivary neoplasms recapitulating a carcinoma with thymus-like elements (CASTLE) have been the subject of recent case reports [60–64]. In the recent largest series, the authors reviewed clinicopathological, immunohistochemical and molecular findings in 20 salivary gland tumors with a thymus-like phenotype (18 histologically benign tumors and two with a malignant component) [65]. Original diagnoses were non-sebaceous adenoma (*n* = 11) and unclassified thymus-like lymphoepithelial neoplasm (*n* = 9). The patients were 13 males and seven females aged 28 to 83 years (median, 61 years). All tumors originated in the parotid gland with a median tumor size of 27 mm. A cystic component was noted in eight cases (40%). Histologically, these tumors were composed of large squamoid cells with indistinct cell borders, forming large irregular branching and anastomosing aggregates within a lymphoid stroma with Hassall corpuscle-like structures and intraepithelial sprinkling of lymphocytes (Fig. 10A-B). All tumors were positive for p63/p40 and CK5/CK14. CD5 (Fig. 10C) and CD117 (Fig. 10D) were expressed in 13/20 (65%) and 15/19 (79%) cases, respectively. The malignant component in two cases showed a lower CD5/CD117 expression. Targeted DNA sequencing revealed pathogenic/likely pathogenic inactivating *CYLD* mutations in 4/7 cases (57%) [65]. Possible inclusion in the next WHO classification remains to be determined.

### Biphasic myoepithelial carcinoma with loss of 5p/5q

In a recent study on DNA methylation of salivary gland tumors Jurmeister et al. identified a group of previously unclassifiable, clinically indolent tumors with striking female predominance [66]. These tumors are characterized by variable admixture of biphasic-tubular and monophasic-myoepithelial areas, low or absent nuclear atypia, and minimal proliferation activity. Frequent invasive behavior, a solitary lymph node metastasis, and the molecular alterations, altogether, strongly support classification as a presumably low-grade carcinoma. In addition to distinct DNA methylation profiling, copy number analysis revealed unique alterations with highly recurrent chromosome 5p/5q loss and frequent amplification of the *MDM2* locus on chromosome 12q.

It has been postulated that this tumor group represents a distinct salivary carcinoma entity rather than a variant of any other existing one. The histomorphologic features are, however, only moderately distinct. Positivity for MDM2 may be very helpful, and DNA methylation analysis and copy number analysis (5p5q) can definitively prove the diagnosis. Provisional designation “Biphasic myoepithelial carcinoma with 5p/5q loss” has been proposed [67].

### Proposed grading systems of selected salivary gland carcinomas

Due to the extraordinary diversity of malignant salivary gland tumors, there is no universal grading system. Among carcinomas with a grading system available are MEC (the current WHO classification lists three different grading systems) and AdCC [1]. Recently, efforts to construct grading systems for other salivary gland carcinomas, specifically for acinic cell carcinoma (AciCC) and secretory carcinoma (SC) have been proposed.

### Grading system for acinic cell carcinoma

A new grading system for AciCC was proposed by Xu et al. [68]. They analyzed a total of 117 cases of AciCC. The group consisted of 63 females and 54 males in the age range of 11–88 years (median, 52 years). All tumors were located in the parotid gland. Data from the follow-up period ranging from 1 to 343 months (median 42 months) were available for 108 patients. Thirty patients developed distant metastases, most commonly in the lungs and bones. The disease-specific survival rates at three, five, and ten years were 88%, 85% and 75%, respectively.

The authors divided all tumors into three grades based on four microscopic parameters (mitotic activity, presence of necrosis, appearance of the tumor margin (well-defined vs. infiltrative) and presence of fibrosis at the tumor periphery): low-grade, intermediate-grade, and high-grade. When analysing the data, it was determined that combining low-grade and intermediate-grade tumors into one group and comparing them with high-grade tumors provided the best prognostication. High mitotic activity (≥ 5 mitoses per 10 HPF) and/or presence of necrosis were indicative of high-grade tumors. High-grade, defined in this way, was shown to be an independent adverse prognostic factor for overall survival (*p* = 0.025). While the overall 5-year survival of patients with low- and intermediate-grade carcinoma was 100%, the overall 5-year survival of patients with high-grade carcinoma was dramatically lower at 50%. Further studies are required to validate this grading scheme.

### Grading system for salivary secretory carcinoma

A new grading system for SC was proposed by Bradová, et. al. [69]. A total of 215 cases of SC were analyzed, including 123 males and 87 females in the age range of 7–94 years (median, 48 years). By far the most common location of the tumor was in the parotid gland (74% of cases). Complete data from the follow-up period were available for 109 patients and ranged from 2–600 months (median, 48 months). Local recurrence occurred in 15% of patients, and only five patients died of the tumor.

The authors divided all tumors based on six microscopic parameters (predominant architecture, pleomorphism, mitotic/proliferative activity, presence of tumor necrosis, lymphovascular invasion, perineural spread) into three grades: low-grade, intermediate-grade, and high-grade. The tumors were graded as follows: grade 1: 45%, grade 2: 42%, and grade 3: 13% of tumors. Compared with low-grade and intermediate-grade tumors, high-grade tumors were characterized by solid architecture, more pronounced hyalinization, infiltrative type of invasion into the surrounding area, more pronounced nuclear pleomorphism, presence of lymphovascular invasion or perineural spread, and a proliferation index Ki-67 higher than 30%. Statistical analysis showed that the proposed grading system is a significant indicator, among other things, for 5-year overall survival (*p* = 0.0001). The 5-year overall survival for grade 1, grade 2, and grade 3 tumors was 98%, 83% and 63%, respectively.

## Summary

Molecular pathology of salivary tumors has seen numerous advances in recent years, allowing better classification of previously heterogenous salivary gland tumor categories. Molecular testing has led to the discovery of novel tumor types, including secretory carcinoma and microsecretory adenocarcinoma, which have been established in the 4th and 5th editions of the WHO classification, respectively. Novel and emerging neoplastic entities are continuously defined by both characteristic molecular alterations and reproducible morphologies. Nevertheless, the synthesis of morphological patterns and the molecular alterations driving them is rarely straightforward. Questions remain concerning the classification of neoplasms with morphologies matching known subtypes but lacking the acknowledged molecular alterations. On the other hand, novel tumor subtypes have been identified based on subtype-specific molecular alterations, even if histomorphologies may be variable. More studies are needed to answer these questions, and possible inclusion in the next WHO classification remains to be determined.

## Data Availability

All data generated or analyzed during this study are included in this published article [and its supplementary information files]. Data supporting the findings of this study are available within the article. The complete datasets generated during and/or analyzed during the current study are available from the corresponding author upon reasonable request.
